# Oxidative Stress Promotes Corticosteroid Insensitivity in Asthma and COPD

**DOI:** 10.3390/antiox10091335

**Published:** 2021-08-24

**Authors:** Brandon W. Lewis, Maria L. Ford, Lynette K. Rogers, Rodney D. Britt

**Affiliations:** 1 Center for Perinatal Research, Abigail Wexner Research Institute at Nationwide Children’s Hospital, Columbus, OH 43205, USA; brandon.lewis@nationwidechildrens.org (B.W.L.); maria.ford@nationwidechildrens.org (M.L.F.); lynette.rogers@nationwidechildrens.org (L.K.R.); 2 Department of Pediatrics, The Ohio State University, Columbus, OH 43210, USA

**Keywords:** oxidative stress, corticosteroids, asthma, COPD

## Abstract

Corticosteroid insensitivity is a key characteristic of patients with severe asthma and COPD. These individuals experience greater pulmonary oxidative stress and inflammation, which contribute to diminished lung function and frequent exacerbations despite the often and prolonged use of systemic, high dose corticosteroids. Reactive oxygen and nitrogen species (RONS) promote corticosteroid insensitivity by disrupting glucocorticoid receptor (GR) signaling, leading to the sustained activation of pro-inflammatory pathways in immune and airway structural cells. Studies in asthma and COPD models suggest that corticosteroids need a balanced redox environment to be effective and to reduce airway inflammation. In this review, we discuss how oxidative stress contributes to corticosteroid insensitivity and the importance of optimizing endogenous antioxidant responses to enhance corticosteroid sensitivity. Future studies should aim to identify how antioxidant-based therapies can complement corticosteroids to reduce the need for prolonged high dose regimens in patients with severe asthma and COPD.

## 1. Introduction

Increased oxidative stress is commonly linked with the pathogenesis and severity of inflammatory lung diseases [1,2,3,4,5]. Oxidative stress develops due to the accumulation of reactive oxygen or nitrogen species (RONS) and/or the loss of antioxidant capacity. RONS contribute to the development of airway inflammation, mucus hypersecretion, airway hyperresponsiveness, and thickening or remodeling, which are hallmark pathological features of asthma and chronic obstructive pulmonary disease (COPD) [6,7,8,9]. Asthma and COPD are associated with several factors that contribute to pathophysiology, including environmental exposures, which induce oxidative stress by damaging the airway epithelium and instigate immune cell infiltration [10,11,12,13]. Functional changes involving airway thickening and stiffening contribute to airflow obstruction and acute exacerbation. These structural and functional alterations are worsened in asthma and COPD patients with corticosteroid insensitivity [14,15,16].

Asthma and COPD adversely affect millions worldwide and remain significant health burdens [17,18]. Asthma is among the leading causes of hospitalization among children who experience respiratory symptoms, including wheezing, coughing, and difficulty breathing [17]. COPD is among the leading causes of death in adults worldwide and is characterized by emphysema, bronchitis, and small airway disease [18,19]. In addition to respiratory symptoms, patients with asthma or COPD are at risk of acute exacerbations, which can be life-threatening events that increase lung morbidity and are potentially fatal [20,21].

Corticosteroids are key anti-inflammatory drugs used to manage symptoms, and corticosteroid sensitivity is commonly used to characterize disease severity and phenotype [19,22,23,24,25,26]. Patients with mild-moderate asthma or COPD have relatively high corticosteroid sensitivity, as inhaled corticosteroids at low doses improve lung function and acute exacerbation frequency [26]. In contrast, patients with severe asthma or moderate-severe COPD require the administration of inhaled or systemic corticosteroids at greater doses with decreased effectiveness, resulting in persistent airway inflammation, airflow obstruction, and more frequent exacerbations [22,27]. The healthcare burden and costs associated with corticosteroid insensitivity are substantial, and patients with moderate-severe asthma or COPD contribute to over 50% of the asthma- and COPD-related healthcare costs [28,29,30]. The prolonged use of systemic, high dose corticosteroids leads to adverse side effects [31], and its cumulative burden complicates disease management [32,33,34,35].

Oxidative stress has been shown to decrease responsiveness to corticosteroids through altering the glucocorticoid receptor (GR) expression and signaling, which is likely one mechanism for corticosteroid insensitivity [36,37]. Several studies show a correlation between oxidative stress and airway disease severity, implicating oxidative stress in corticosteroid insensitivity [38,39,40]. In the present review, we discuss oxidative stress as a key driver of pro-inflammatory responses that promote airway inflammation in the presence of corticosteroids. The importance of this mechanism is highlighted by accumulating evidence that oxidative stress can disrupt glucocorticoid receptor (GR) activity. We also discuss the potential for strategies that stimulate endogenous antioxidant responses in the airway to enhance corticosteroid sensitivity.

## 2. Corticosteroid Insensitivity in Asthma and COPD Pathophysiology

Chronic airway inflammation is key for the development of structural and functional changes to the airway that restrict airflow and contribute to exacerbations in asthma and COPD [41]. The inflammatory milieu in the airway is complex, heterogenous, and possibly dynamic. This complexity and the broad anti-inflammatory properties of corticosteroids make their use appealing to manage symptoms and disease progression. Studies in asthma or COPD patient cohorts have identified different physiological and immunological responses that are thought to influence disease severity and corticosteroid sensitivity [42,43].

### 2.1. Airway Inflammation

T helper 2 (Th2) Inflammation. The adaptive Th2 immune response is the predominant immune phenotype in allergic asthma and is observed in the majority of pediatric and adult patients with mild-moderate asthma [44]. These patients exhibit increases in Th2 effector cytokine levels (IL-4, IL-5, IL-13) that are produced by the CD4+ Th2 cells and group 2 innate lymphoid cells (ILC2). While IL-5 is the main driver of eosinophil recruitment, IL-4 and IL-13 promote mucous cell metaplasia, airway hyperresponsiveness, and remodeling [45]. Corticosteroid sensitivity in patients with Th2 inflammation is variable, with some having high sensitivity while others with more severe disease have moderate to low sensitivity.

Th1 and Th17 Inflammation. For COPD, Th1 and/or Th17 inflammation is the predominant adaptive immune response. However, the same responses are also present in patients with severe asthma [46,47,48]. The presence of Th1 and Th17 inflammation is associated with more severe disease and reduced corticosteroid sensitivity [23,27,46,49]. Th1 and Th17 adaptive immune responses are characterized by the infiltration of Th cells producing IFNγ and IL-17A, respectively. These adaptive immune phenotypes are often associated with responses to lung injury or infection. Increases in Th1 and Th17 inflammation are accompanied by neutrophil infiltration, which correlates with corticosteroid insensitivity [50,51]. IFNγ and IL-17A induce pro-inflammatory responses in other immune cells, airway epithelium, and smooth muscle to promote neutrophil airway infiltration, airway hyperresponsiveness, and remodeling [49,52,53].

### 2.2. Airway Structure and Function

Airway epithelium. The airway epithelium is a layer of cells that lines the airway lumen and is responsible for maintaining an innate barrier to airborne debris and pathogens [54]. The airway epithelium is composed of ciliated cells that are responsible for removing airborne pathogens and mucus away from lower airways and goblet cells that secrete mucus. In asthma and COPD, the airway epithelial barrier integrity is compromised with increased mucus production and accumulation, resulting in structural changes and airflow obstruction [55,56]. In addition to airway structure, the airway epithelium also contributes airway inflammation releasing pro-inflammatory cytokines such as TNFα and IL-33, which contribute to Th1 and Th2 inflammation, respectively [57,58]. Corticosteroids inhibit pro-inflammatory responses in the airway epithelium and preserve epithelial integrity upon injury by infection or environmental insult [59,60,61]. Airway mucus production and epithelial integrity remain unaffected by corticosteroid treatment in severe asthma and COPD [59,62].

Airway Smooth Muscle (ASM) and Fibroblasts. Persistent airway thickening and remodeling, which are hallmark pathological features in asthma and COPD, contribute to airflow obstruction and impaired lung function [14,63,64,65]. Increases in ASM and airway fibroblasts in the sub-epithelial layer can be attributed to increases in proliferation, extracellular matrix deposition, and hypertrophy [66,67,68,69,70]. Airway hyperresponsiveness is a functional characteristic that affects airway tone, acute exacerbations, and contributes to airway narrowing in response to bronchoconstrictors such as histamine [68]. Airway inflammation augments ASM Ca^2+^ responses and hypercontractility that lead to airway hyperresponsiveness, contributing to poor lung function and exacerbations [71]. Studies show these structural and function characteristics in airway disease are largely unresponsive to corticosteroids, worsening airway structure overtime [72,73,74].

## 3. Factors Contributing to Oxidative Stress

Reactive oxygen and nitrogen species (RONS) are critical for the homeostatic cellular and physiological functions in the lung. Within a pro-oxidant and pro-inflammatory environment, RONS accumulation has numerous detrimental consequences on cellular metabolism, tissue damage, and, ultimately, cell death [75]. Macromolecules such as proteins and lipids are highly susceptible to oxidative reactions that modify molecular structure and function. In asthma and COPD, RONS (e.g., superoxide, hydrogen peroxide, hydroxyl radicals, and nitrogen dioxide) levels are found to be increased in the plasma, sputum, and bronchoalveolar lavage tissue. This is illustrated by the substantial increases in the levels of oxidative stress biomarkers, including malondialdehyde (MDA), thiol oxidation, protein carbonyls, oxidized fatty acids, and exhaled nitric oxide (FeNO) [76,77].

**Figure 1:**
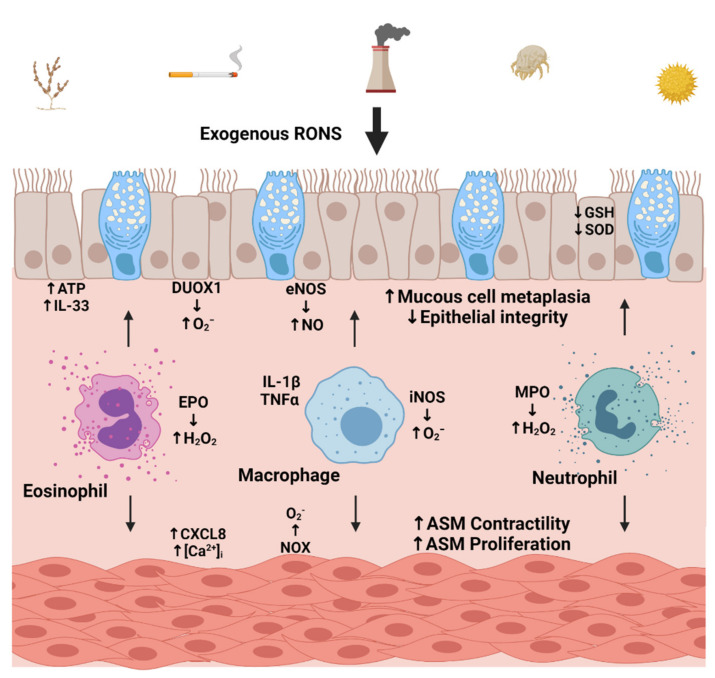
Effects of oxidative stress on airway inflammation. Environmental allergens, cigarette smoke, pollution, and pathogens interact with the airway epithelium, leading to increases in exogenous or endogenous reactive oxygen and nitrogen species (RONS) levels and contributing to the pathogenesis of asthma and COPD. In response, the airway epithelium generates superoxide (O_2_^−^) and nitric oxide (NO) and releases ATP and IL-33. Similarly, airway smooth muscle (ASM) release pro-inflammatory mediators such as CXCL8 and superoxide. In addition to loss in barrier integrity, the airway epithelium exhibits mucous cell metaplasia. ASM becomes hypercontractile and proliferates, leading to hypercontractility and airway thickening. Further, inflammatory cells such as eosinophils and neutrophils release eosinophilic peroxidase (EPO) and myeloperoxidase (MPO), respectively, in response to increased intracellular oxidative stress. EPO and MPO further contribute to increases in airway oxidative stress by generating hydrogen peroxide (H_2_O_2_). Increased iNOS activity in macrophages also contributes to RONS and the release of inflammatory mediators. The activity of antioxidant mechanisms, such as the glutathione (GSH) system and superoxide dismutase (SOD), are dysfunctional and are found to be reduced in airway epithelial cells in severe asthma and COPD, further enhancing oxidative stress and airway inflammation. Created with “Biorender.com”. (**↑**) denotes increased levels and (↓) denotes decreased levels.

Patients with severe asthma and COPD often exhibit greater levels of oxidative stress biomarkers, with increased levels correlating with worsened symptoms, decreased lung function, and corticosteroid insensitivity [37,75,78,79,80,81,82,83]. This oxidative burden is achieved by exposure to environmental and cellular sources of RONS. Both immune and airway structural cells respond and contribute to increased oxidative stress in the lung. Innate immune cells, e.g., macrophages, neutrophils, and eosinophils, produce RONS as a consequence of activation during pro-inflammatory responses (Figure 1) [75,84]. In airway structural cells, mitochondria generate RONS to enhance oxidative stress and contribute to airway inflammation [85,86,87].

### 3.1. Environmental Sources

Allergens. Indoor and outdoor allergens are sources of proteases that damage the airway epithelium and induce robust innate and adaptive immune responses [88,89]. Allergens such as house dust mite (HDM), dander, pollen, and fungal allergens contribute heavily to the pathogenesis of asthma in both children and adults [90,91]. Allergens, such as HDM, can induce Th2-mediated inflammatory responses, resulting in cellular injury and destruction and ultimately the disruption of the airway epithelial barrier [88]. In asthma, oxidative stress and DNA damage are generated following the sensitization and challenge to HDM [92]. Allergen sensitization is increasingly recognized as a factor that can affect asthma severity and corticosteroid sensitivity, particularly in individuals sensitized to fungal allergens [90,91].

Smoking. Exposure to cigarette smoke is a leading factor in the development of COPD with increases in lung inflammation. Smoking can also induce acute exacerbations that are associated with a decreased sensitivity to corticosteroids [78,93,94,95,96]. With its toxic chemicals, chronic exposure to cigarette smoke damages lung epithelial barriers and initiates pro-inflammatory responses with increased immune cell infiltration and cytokine release [97,98]. Components of cigarette smoke readily increase RONS production in mitochondria, resulting in increased oxidative stress with the significant oxidation of proteins and lipids [99,100]. Smoking-mediated damage to the airway and alveolar compartments is largely unaffected by treatment with corticosteroids [101], making cigarette smoke an important factor in corticosteroid insensitivity.

Air pollution. Air quality is an important factor that is affected by ozone and particulate matter concentrations. Increases in pollutant levels contribute to disease pathogenesis and exacerbations in asthma and COPD [80,81,102]. Ozone is an oxidant that induces Th17-mediated neutrophilic airway inflammation and is associated with decreased corticosteroid sensitivity [79,103,104]. Exposure to other environmental pollutants, such as diesel exhaust and <2.5 µm particulate matter (PM_2.5_), also induces high levels of oxidative stress in the lung [82,83,105]. Similar to ozone, diesel exhaust induces Th17 inflammation with increases in IL-17A levels and neutrophil infiltration [106,107]. Airway inflammation and hyperresponsiveness remain increased in diesel exhaust-exposed mice treated with corticosteroids [108]. Although PM_2.5_ is known to increase airway inflammation and augment allergic responses [109,110], its impact on corticosteroid sensitivity remains poorly understood.

### 3.2. Cellular Sources of Oxidative Stress

Macrophages. Lung macrophages play a pivotal role in asthma and COPD [111,112]. Macrophages are abundant in the lung and generate RONS to kill invading pathogens [113,114,115]. In respiratory burst, increased inducible nitric oxide synthase (iNOS) and NAPDH oxidase activity in macrophages results in increased hydrogen peroxide, nitric oxide, superoxide, and peroxynitrite production [116,117]. Increased RONS levels in lung macrophages lead to greater pro-inflammatory cytokine release [11]. RONS also affect lung macrophage function, reducing their ability to phagocytize pathogens and apoptotic cells, which is an important process [118]. Persistent oxidative stress in lung macrophages may contribute to reduced corticosteroid sensitivity in severe asthma and COPD [119,120].

Eosinophils. Following lung infiltration and activation during allergic responses, eosinophils release eosinophilic extracellular traps (EETs) that contain eosinophilic peroxidase (EPO), releasing hydrogen peroxide [121,122]. Pharmacological studies suggest that EET formation and EPO activity are dependent upon the generation of RONS and oxidative stress [123]. Conversely, recent studies show that hydrogen peroxide can also contribute to eosinophil apoptosis, an important mechanism in the resolution of allergic responses in the lung [124]. Corticosteroids are largely effective at reducing airway eosinophilia, but in severe asthma, greater doses are required to reduce their levels in circulation and the lung [125,126].

Neutrophils. Neutrophil lung infiltration and the expression levels of chemoattractants, CXCL1 and CXCL8, are increased in patients unresponsive to corticosteroids, implicating neutrophils in corticosteroid insensitivity in asthma and COPD [127,128,129,130,131]. Increased neutrophils in the lung can be attributed to their enhanced survival [132], which contributes to increased oxidative stress from myeloperoxidase (MPO)-mediated oxidative burst [84]. MPO produces hydrogen peroxide and is part of neutrophil extracellular traps (NETs), which are composed of antimicrobial proteins and enzymes. While NETs are needed for the neutrophil clearance of pathogens, they can also contribute to oxidative stress and persistent lung inflammation [133,134,135]. Neutrophils isolated from patients with severe asthma produced higher NET levels, suggesting that NETs may contribute to corticosteroid insensitivity [132,136,137]. Pham et al. concluded that the release of NETs induced the cell death of human airway epithelial cells, while treatment with NET and MPO antibodies increased epithelial cell survival [136]. In summary, these data highlight the role of neutrophils in airway inflammation and the severity of airway disease.

Airway epithelium. Airway injury results in the loss of epithelial barrier integrity. As a result, airway epithelial cells generate a substantial amount of RONS, including nitric oxide (NO) and chemoattractants that recruit immune cells to the airway [138]. Mitochondria are an important source of energy through ATP production. In the context of cell injury and mitochondrial dysfunction, ATP can be released into extracellular spaces and function as a damage associated molecular pattern molecule (DAMP). ATP can induce pro-inflammatory responses in the surrounding tissue through P2X/2Y receptor-mediated responses [85,139]. In asthma and COPD, ATP levels are increased in bronchoalveolar lavage fluid and correlated with disease severity, implying that ATP may be an important epithelial-derived DAMP that contributes to inflammatory responses in airway disease [140,141,142,143].

Intracellular RONS contribute to increased IL-33 release, which is a key mechanism for initiating Th2-mediated inflammation in asthma [10]. IL-33 was also found to be increased in mice exposed to cigarette smoke, implicating a role in COPD [144]. IL-13, another key cytokine in Th2 inflammation and asthma, induces airway epithelial cell superoxide production through the NADPH oxidase, DUOX1, during allergen challenges [145]. Overall, the airway epithelium is an important source of RONS and secretes pro-inflammatory mediators that are regulated by oxidative stress.

Airway Smooth Muscle (ASM). Upon pro-inflammatory cytokine stimulation, intracellular RONS contribute to increased ASM contractility, proliferation, and pro-inflammatory cytokine/chemokine release [146,147,148]. Intracellular Ca^2+^ ([Ca^2+^]_i_) regulation is central to how ASM responds to pro-inflammatory cytokines, and its regulation by mitochondria is emerging as an important mechanism [68,85]. Recent studies show that ozone exposure decreases mitochondrial membrane potential and RONS generation to contribute to airway hyperresponsiveness and increased ASM mass [4]. Mitochondria can contribute to [Ca^2+^]_i_ through membrane-bound Ca^2+^ channels, which, during exposure to TNFα, leads to Ca^2+^ efflux from the mitochondria to the cytosol [149]. Increases in oxidative stress and [Ca^2+^]_i_ also enhance ASM proliferation, which contributes to increased ASM mass and airway thickening [150]. Targeting antioxidants to the mitochondria reduces ASM proliferation and CXCL8 release [4], highlighting the importance of mitochondria in ASM dysfunction during airway inflammation. Although RONS are known to affect ASM responses to airway inflammation, little is known about the release of RONS by ASM and the effects on the surrounding cells. Furthermore, impact of mitochondrial dysfunction during oxidative stress conditions on corticosteroid sensitivity in ASM remains largely unexplored.

## 4. Oxidative Stress Promotes Corticosteroid Insensitivity

Corticosteroids reduce inflammation by binding to the glucocorticoid receptor (GR) in the cytosol and stimulating GR to translocate to the nucleus, where it regulates gene expression [36]. Within the context of airway disease, GR affects several genes and pathways associated with inflammation and metabolic processes [151]. The current model by which GR is thought to suppress inflammation involves modulating chromatin structure, suppressing promoter activity at pro-inflammatory genes, and enhancing the expression of anti-inflammatory mediators [152,153]. The wide-ranging anti-inflammatory effects of corticosteroids are centered on their ability to modulate gene expression in multiple cell types, including immune cells, epithelium, smooth muscle, and fibroblasts. However, recent studies show that the effects of GR are cell-type dependent [154,155], which is an important factor that may influence corticosteroid sensitivity in asthma and COPD.

### 4.1. Disruption of Glucocorticoid Receptor (GR) Signaling

GR Expression. Oxidative stress has been implicated in contributing to corticosteroid insensitivity by affecting GR signaling and activity [59]. The expression of the active isoform that mediates corticosteroid anti-inflammatory effects, GRα, is reduced in the lungs of patients with severe asthma and COPD [156]. Immune and airway epithelial cells isolated from patients with corticosteroid insensitivity show an increased expression of GRβ, a dominant negative isoform that is unable to induce anti-inflammatory responses [157,158]. Similarly, impaired GR nuclear translocation has been observed in both immune and airway structural cells from patients with asthma and COPD [159,160,161]. Oxidative stress reduces GRα expression and impairs GR DNA binding activity in airway epithelial and smooth muscle cells, in vitro [162,163,164]. These studies highlight the negative impact of oxidative stress on GR signaling through a reduction in GRα expression.

GR Phosphorylation. Post-translational modifications on GR, notably phosphorylation, are important for GR activity. GR phosphorylation regulates nuclear translocation and DNA binding activity, influencing corticosteroid sensitivity [165,166,167]. On human GR, Ser211 phosphorylation is important for DNA binding activity, while Ser226 phosphorylation impairs GR nuclear translocation [168,169]. These residues are among multiple phosphorylation sites within GR that are targeted by several kinases and phosphatases, affecting GR activation and its regulation of gene expression [170,171]. Oxidant sensitive mitogen activated protein kinase (MAPK) pathways, p38 and JNK, target and phosphorylate Ser226, leading to reduced GR nuclear translocation and GR activity [172,173,174,175]. The inhibition of p38 improves corticosteroid sensitivity in immune and structural epithelial cells isolated from severe asthmatics and allergen challenged mice with severe allergic airway inflammation [173,176,177,178]. In ASM, exposure to pro-inflammatory cytokines increases protein phosphatase 5 expression, which reduces Ser211 phosphorylation and GR activity [167,179]. These studies highlight the impact of oxidative stress on GR phosphorylation and activity through its modulation of MAP kinases.

GR and HDAC2. In severe asthma and COPD, histone deacetylase 2 (HDAC2) expression and activity are reduced, resulting in the deacetylation of GR (Lys494 and Lys495) and reduced HDAC2 activity at histones associated with pro-inflammatory genes [180,181,182,183]. HDAC2 is recognized as an important factor for the anti-inflammatory effects of corticosteroids. Oxidative stress generated by cigarette smoke leads to PI3Kδ activation, which phosphorylates HDAC2, reducing its activity and corticosteroid sensitivity [184]. The role for PI3K is further highlighted in allergen-challenged mice, where miR-21 antagonizes PTEN, a negative regulator of PI3K, to reduce HDAC2 activation. Mice treated with miR-21 antagomir or PI3K pharmacological inhibition demonstrated increased HDAC2 expression and improved corticosteroid insensitivity [185].

Several strategies have been identified to restore HDAC2 activity and corticosteroid sensitivity in asthma and COPD [37]. Theophylline, a nonspecific phosphodiesterase (PDE) inhibitor and bronchodilator, was identified to improve HDAC2 expression in COPD, which led to examining its potential to improve corticosteroid sensitivity in patients with COPD. While early studies showed increased HDAC activity and reduced inflammation in COPD patients treated with corticosteroids and low-dose theophylline [186,187,188], larger clinical studies showed no improvement in lung function or exacerbation frequency [189].

### 4.2. Oxidative Stress and Pro-Inflammatory Signaling

MAP Kinase and NFκB pathways. Oxidative stress augments pro-inflammatory signaling that contributes to poorly controlled inflammation and corticosteroid insensitivity [190]. Increases in RONS within the lung are associated with increased pro-inflammatory cytokine expression [191]. Hydrogen peroxide increases CXCL8 secretion, which is insensitive to the corticosteroid, budesonide [59]. Under oxidative stress and cellular injury, RONS can directly activate p38 and JNK, which subsequently leads to the activation of key pro-inflammatory transcription factors, namely, nuclear factor κB (NFκB) and activator protein-1 (AP-1) [192,193,194]. NFκB activation by oxidants enhances inflammatory cytokine expression and oxidative stress in asthma [195]. AP-1 is a heterodimer that is essential for the transcription of many immune, inflammatory, and antioxidant genes such as γ-glutamine-cysteine ligase (γ-GLCL) but can also contribute to the activation of second messengers to further propagate inflammation [193,196]. Despite being important targets of corticosteroids and GR signaling, NFκB and AP-1 activation are found to be higher in patients with asthma and COPD [197,198,199]. In poorly controlled asthma, NFκB activation remains elevated, suggesting that its activation may be insensitive to corticosteroids in individuals with more severe airway disease [200].

**Figure 2:**
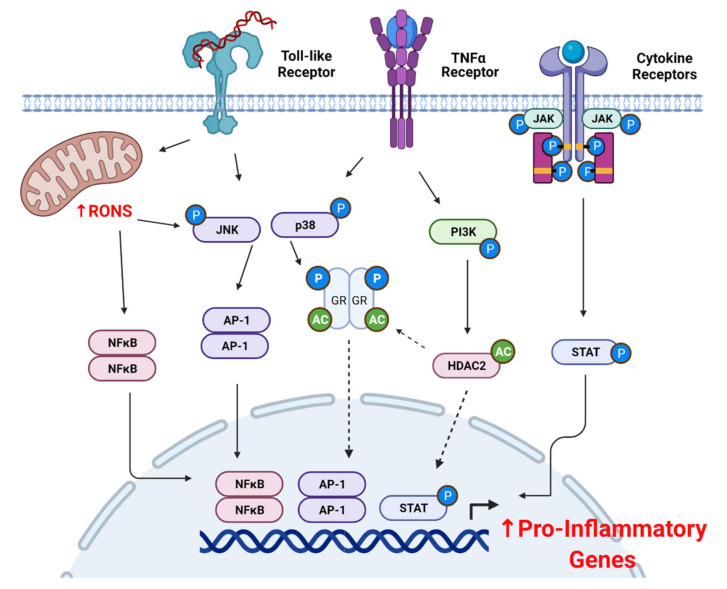
Reactive Oxygen Nitrogen Species (RONS) and Glucocorticoid receptor (GR) Signaling. The activation of pro-inflammatory signaling pathways (Tumor necrosis factor Receptor (TNFR), Toll-like Receptor, and Cytokine Receptors) leads to increases in RONS production in mitochondria and cytosol. Posttranslational modification such as acetylation and phosphorylation affect GR and HDAC2 activity, leading to augmented pro-inflammatory responses. Dashed arrows represent decreased activity and function. The figure was adapted from “B Regulatory Cell Surface Receptor Influences IL-10-mediated Immune Tolerance” (2021). Retrieved from https://app.biorender.com/biorender-templates (accessed on 27 July 2021).

JAK/Stat pathways. The binding of specific cytokines and interferons to their receptors leads to the transphosphorylation of tyrosine residues on Janus Kinases (JAK) [201]. The activation of JAK in turn recruits and phosphorylates the tyrosine residues of signal transducers and the activators of the transcription (STAT) family of signaling molecules. JAK/STAT activation is enhanced in response to elevated levels of H_2_O_2_ or GSH depletion and inhibited by antioxidant treatment [202]. Moreover, activated STAT1 is found at high levels in the airway epithelium of asthmatics, with increased IL-4 and IFNγ expression as contributors to STAT1 activation [203]. We recently observed corticosteroid insensitivity in human airway smooth muscle cells treated with both TNFα and IFNγ, while GR expression, phosphorylation, and activity was maintained [204]. This was found to involve augmented NFκB and JAK/Stat1 signaling pathways that were potentially unresponsive to negative regulatory mechanisms induced by corticosteroids [205]. These findings support the theory that pro-inflammatory signaling pathways can interact to override negative regulatory signals induced by corticosteroids (Figure 2) [204]. The positive feedback loop between ROS production, oxidative stress, and persistent inflammation may offer a novel therapeutic target for the control of severe asthma and COPD.

Alarmins. Damaged or dying cells release alarmins and damage associated molecular patterns (DAMPs) as distress signals for innate immune responses to initiate repair mechanisms [206,207]. Compromised by oxidative stress, airway epithelial cells are injured and lose their integrity, becoming a primary source of alarmins and DAMP in asthma and COPD [207]. Thymic stromal lymphopoietin (TSLP), a key alarmin in asthma pathogenesis, initiates Th2 responses that promote allergic airway inflammation [191,208] and are implicated in contributing to corticosteroid insensitivity in asthma [209,210,211]. Additionally, airway epithelial cells from COPD donors produce greater TSLP levels upon inflammatory stimulation, which was found to be insensitive to dexamethasone [212]. In ASM, cigarette smoke exposure increased TSLPR expression and augmented the pro-inflammatory effects of TSLP on ASM [213]. TSLP has recently emerged following phase 3 clinical trials as a promising therapeutic target for severe asthma [214]. However, its potential for COPD remains unclear.

NLR family pyrin domain containing 3, (NLRP3). Inflammasomes are multiprotein complexes that play an essential role in innate immunity by sensing pathogens and injury and directing the maturation of inflammatory cytokines [215]. The best studied inflammasome, NLRP3, consists of the NLRP3 receptor, an adapter protein, ASC, and caspase-1. Upon stimulation, procaspase-1 is recruited, cleaved, and bound to the receptor via ASC [216]. The assembly of NLRP3 can be dependent on ROS, ion flux, DAMPs, cytokines, or ATP. Once active, NLRP3 can further influence oxidative stress by enhancing the production of proinflammatory mediators and ROS. A primary function of the NLRP3 is the cleavage of the precursors of IL-1β and IL-18 to their active forms and their subsequent release into the extracellular space, where they promote inflammation [216,217,218]. IL-1β levels are higher in the serum and induced sputum of symptomatic asthmatics than in asymptomatic patients [219], while in rodent models of asthma, IL-1β is increased and contributes to airway inflammation [220,221]. Recent studies demonstrated an important role NLRP3 and IL-1β play in mediating neutrophil infiltration and corticosteroid insensitivity in a mouse model of severe allergic airway inflammation [222]. Overall, these data highlight the redox-sensitive NLRP3 inflammasome as a key mechanism that contributes to corticosteroid insensitivity.

## 5. Targeting Oxidative Stress to Improve Corticosteroid Sensitivity

The cell protects itself from injury caused by oxidative stress through an extensive network of enzymatic and non-enzymatic molecules, collectively called antioxidants. Antioxidants function by maintaining a physiological balance between the generation of RONS and their removal [75,223]. Imbalances in oxidant/antioxidant status are causally linked to asthma and COPD pathophysiology such as airflow obstruction, airway hyperreactivity, and remodeling [75,112]. Moreover, the severity of airway disease is directly correlated with the amount of RONS generated [224,225,226]. In addition to greater quantities of RONS produced in the lungs of asthmatic patients, the levels of antioxidants such as superoxide dismutase (SOD) and catalase are lower than those found in healthy lungs [227], and diminished antioxidant capacities likely contribute to corticosteroid insensitivity [37,228].

### 5.1. Key Antioxidant Systems

Glutathione (GSH). GSH is a major non-enzymatic antioxidant in the lung and is synthesized intracellularly from the amino acids cysteine, glycine, and glutamate by the activity of γ-glutamyl ligase (rate limiting) and glutathione synthetase [229]. Approximately 90% of all lung GSH is maintained in the reduced form, and epithelial lining fluid contains roughly 50 times more reduced GSH than plasma (300 µM) [230]. Mechanistically, glutathione peroxidases (GPXs) use the reducing equivalents of GSH to reduce cellularly generated hydrogen peroxide to H_2_O, changing GSH to its oxidized form glutathione disulfide (GSSG). GSSG is then reduced back to GSH by the activity of glutathione reductase and by reducing equivalents from NADPH [231]. In addition to the endogenous production of GSH and the reduction of GSSG, the rapid import of GSH into the cell and the export of GSSG from the cell facilitates overcoming oxidant stress and the accumulation of GSSG [232,233]. Thus, a pool of reduced GSH is essential for maintaining the cell in a principally reduced state and the cellular components, such as proteins and enzymes, in a functional configuration [231].

Overall, GSH levels are lower in the serum and lung cells of children and adults diagnosed with asthma [29,234]. GSH levels are also lower in the exhaled breath condensates of children with severe asthma than in healthy children. However, the levels are higher in lung lining fluids [234]. More significantly, in children with asthma and adults with COPD, glutathione levels in exhaled breath were lower than control, and these levels increased with oral steroid treatment [235,236]. In mice, GSH depletion augments allergic airway inflammation through increased p38 activation and iNOS activity [237]. These studies highlight the importance of glutathione in maintaining redox balance in the airway.

Superoxide Dismutases (SODs). SODs are responsible for catalyzing the reaction of superoxide to hydrogen peroxide [238]. There are three isoforms of SOD in mammals, copper-zinc (CuZn)SOD, manganese (Mn)SOD, and extra-cellular (EC)-SOD [2]. CuZnSOD accounts for about 80–90% of the intracellular SOD activity and is located primarily in the cytosol [239]. MnSOD makes up approximately 10% of the intracellular SOD activity and while it is initially expressed in the cytosol, it is imported into the mitochondria and is located primarily in the mitochondrial matrix [2,239]. EC-SOD is the secretory and extracellular form. It is found in the interstitial space of the lungs, primarily surrounding blood vessels and airways [238,240].

SODs are present in every mammalian cell, but lower expression levels and activity are observed in the lung lining fluids and airway epithelial cells of individuals with asthma and COPD compared to healthy controls [241,242]. A loss of activity can occur within minutes of an acute response and is related to modifications of the SOD protein that impairs enzymatic function [243]. Specifically, CuZnSOD is inactivated by the oxidation of critical histidine residues, while MnSOD and ECSOD are inactivated through the chlorination and nitration of tyrosine residues, respectively [75,244,245]. These modifications are a contributing factor to the increase in RONS, increased oxidant stress, and ultimately increased airway hyperreactivity and remodeling during asthma exacerbation [225,241,246,247].

Catalase. Catalase is an oxidoreductase that works in concert with GPX to neutralize hydrogen peroxide. While catalase is effective on small molecules and at high concentrations, prolonged oxidant stress causes catalase activity to decrease [248]. This reduction is due to modifications to the tyrosine residues found in the lungs of asthmatics, similar to that of SOD [249,250]. Although catalase is considered a first line of defense against hydrogen peroxide, its effectiveness in chronic oxidant stress is limited. Inactivation of this important antioxidant mechanism likely contributes to persistent airway inflammation and corticosteroid insensitivity.

Thioredoxin (TRX). TRX is an oxidoreductase that contains a dithiol-disulfide active site [75,251]. Much like glutathione, TRX is maintained in the reduced state by the activity of thioredoxin reductase and the reducing equivalents of NADPH. There are two isoforms of TRX: TRX1, located primarily in the cytosol, and TRX2, located primarily in the mitochondria [252]. In the context of asthma and COPD, TRX has been shown to have a protective role [253]. Allergen-challenged mice treated to enhance TRX1 expression levels exhibited reduced eosinophil lung recruitment, mucous cell metaplasia, and airway remodeling [254,255,256]. Similarly, enhanced TRX1 expression protects mice from cigarette smoke-induced lung inflammation and emphysema [257,258,259]. Less is known about TRX expression and activity in patients with asthma and COPD. Levels of serum TRX1 were found to be increased during an asthma exacerbation and inversely correlated with lung function [260]. Conversely in COPD acute exacerbation, TRX1 and TRXR1 expression is reduced in serum samples with increased levels of 4-hydroxy-2-nonenal (4HNE)-protein adducts. The role of the TRX system in asthma and COPD is not clear, particularly in the context of more severe phenotypes and corticosteroid sensitivity.

Nuclear factor erythroid 2-related factor 2 (Nrf2). Nrf2 is a transcription factor that regulates the expression of genes associated with protecting the cells from oxidant stress and damage [261,262]. Nrf2 is tethered in the cytosol by its interaction with Keap1 but is released by the presence of oxidative stress to translocate to the nucleus, bind to the antioxidant response elements, and activate the expression of genes involved in endogenous antioxidant responses [38]. Nrf2 directly regulates more than 500 genes, including those part of the GSH and TRX systems [261,262]. The antioxidant program regulated by Nrf2 is critical to maintaining lung homeostasis, tolerating oxidant-induced lung injury, and is important for limiting pro-inflammatory responses [261]. Nrf2 knockout mice challenged with allergens and cigarette smoke develop greater airway inflammation that includes increases in leukocyte infiltration and cytokine levels [263,264,265,266]. Further, the genetic deletion of Keap1 protects mice from oxidative stress and inflammation during exposure to acute cigarette smoke [267].

In severe asthma and COPD, Nrf2 expression and activity are substantially reduced, contributing to pronounced oxidative stress and inflammation [268,269,270]. Altered Nrf2 expression and post-translational modifications are implicated in its reduced activity in children with severe asthma [38]. Nrf2 protein levels and Nrf2-mediated antioxidant responses are reduced in asthmatic airway smooth muscle cells compared to non-asthmatics, suggesting that Nrf2 mechanisms are also important for airway hyperresponsiveness and remodeling [271]. The critical roles it plays in lung inflammation and the regulation of endogenous antioxidant responses make Nrf2 an appealing target for therapies in asthma and COPD.

### 5.2. Endogenous Antioxidant Response and Corticosteroid Sensitivity

Targeting oxidative stress and improving redox balance in the airway could improve corticosteroid sensitivity. Advancements in understanding antioxidant mechanisms and their pharmacological targeting may provide an opportunity to improve corticosteroid sensitivity, reducing the burden of high dose corticosteroid while retaining the broad anti-inflammatory effects. In preclinical animal models of asthma and COPD, the administration of antioxidants or the stimulation of endogenous antioxidant responses both reduce airway inflammation and improve lung function. Recent studies also suggest that improving the redox balance in the lung can improve corticosteroid sensitivity [37]. In recent years, several candidate antioxidant strategies have been identified, and some have been tested in combination with corticosteroids (Table A1).

Vitamins C and E. Fruits and vegetables are rich in vitamins and other molecules that serve as antioxidants or important cofactors for antioxidant enzyme reactions. Vitamins C and E scavenge and eliminate oxidation by RONS, particularly membrane lipids [272]. Vitamin C has been shown to reduce airway inflammation, remodeling, and oxidative stress in a model of allergic airway inflammation [273,274]. Similarly, the vitamin E isoform, γ-tocotrienol, was shown to reduce house dust mite-induced allergic airway inflammation through the inhibition of NFκB and increased Nrf2 activation [275]. In humans, γ-tocopherol supplementation was recently shown to reduce eosinophil and neutrophil infiltration in patients with asthma [276]. In mice exposed to cigarette smoke, γ-tocotrienol reduced lung inflammation and enhanced endogenous antioxidant responses. The effects of γ-tocotrienol on inflammation were comparable to treatment with corticosteroids. However, γ-tocotrienol also reduces levels of oxidative stress markers [277].

Polyphenols. Polyphenols and flavonoids are also found in various fruits and vegetables and demonstrate antioxidant activity. Compounds, such as resveratrol and quercetin, have been shown to reduce lung inflammation and oxidative stress while increasing endogenous Nrf2 activation [278,279,280,281,282]. In primary human airway epithelial cells, curcumin was shown to increase HDAC2 expression and improve corticosteroid sensitivity in cells exposed to cigarette smoke extract [283]. Enhanced corticosteroid sensitivity was also observed in ovalbumin challenged mice treated with tetrahyrdocurcumin, suggesting that polyphenols have the potential to improve the corticosteroid efficacy. Despite these observations, the impact of dietary antioxidants on corticosteroid sensitivity needs further exploration to be considered as steroid-sparing treatments in asthma and COPD.

Thiol Supplementation. The administration of thiols to alleviate oxidative stress in chronic lung diseases has been extensively explored. Their ability to scavenge RONS and enhance GSH levels helps protect cells and maintain a healthy redox balance. In a mouse model of corticosteroid resistant asthma, treatment with N-acetylcysteine (NAC) reduced airway inflammation to a greater extent than dexamethasone [284]. NAC increases GSH levels, which are correlated with improved lung function in COPD patients [285,286]. Carbocysteine, a mucolytic agent with antioxidant and anti-inflammatory effects, has been shown to improve corticosteroid sensitivity by increasing HDAC2 expression and GSH levels [287,288]. Although these approaches have been evaluated in clinical studies, their viability as a therapeutic strategy remains limited.

Nrf2 Agonists. Given its broad effects on antioxidant responses, targeting Nrf2 directly is an appealing strategy to suppress oxidative stress and improve corticosteroid sensitivity in severe asthma and COPD. Natural Nrf2 agonists such as sulforaphane have demonstrated efficacy in experimental models of asthma by enhancing antioxidant responses and protecting epithelial tight junction proteins [266]. In COPD models, sulforaphane promotes a robust antioxidant response, inhibits lung inflammation, and protects from cellular damage [289,290,291]. Treatment with sulforaphane improves corticosteroid sensitivity in mice challenged with cockroach allergen extract or exposed to cigarette smoke [292,293]. These effects were absent in Nrf2 knockout mice, suggesting that sulforaphane requires Nrf2 to enhance corticosteroid sensitivity [293]. Further, recently developed Nrf2-enhancing drugs such as RTA-408 have reduced airway hyperresponsiveness, oxidant stress, and inflammatory responses in mouse models of asthma [294]. Although targeting Nrf2 with specific agonists has potential in pre-clinical models, these agonists have yet to demonstrate significant efficacy in patients with asthma or COPD [295,296,297].

H_2_S. Hydrogen sulfide (H_2_S) is a gasotransmitter endogenously generated within cells and has been shown to have several physiological effects [298,299]. H_2_S enhances antioxidant responses by acting as a RONS scavenger and increasing GSH levels in the mitochondria [300,301,302]. Studies show that H_2_S donors can inhibit lung inflammation and increase antioxidant responses [303]. However, its effects in asthma and COPD models remain poorly understood. In alveolar macrophages isolated from patients with COPD, a H_2_S donor was shown to enhance the efficacy of dexamethasone to inhibit IL-8 and TNFα production [304]. Although roles for H_2_S in airway disease are emerging, its ability to enhance endogenous antioxidant responses in the lung provides an additional target to improve corticosteroid sensitivity in asthma and COPD.

## 6. Conclusions

Corticosteroids will likely remain the standard of care, as their unique ability to effectively reduce inflammation in immune cells and airway structural cells make them suitable for managing asthma and COPD. As we have discussed, oxidative stress is a significant contributor to airway inflammation and promotes corticosteroid insensitivity by disrupting GR signaling and augmenting pro-inflammatory responses. Optimal corticosteroid efficacy may be dependent upon maintaining low levels of oxidative stress [226], highlighting the need to develop strategies that can preserve homeostatic redox balance in patients receiving corticosteroids.

Pre-clinical asthma and COPD models clearly demonstrate that enhancing endogenous antioxidant responses, particularly those mediated by Nrf2, can further enhance corticosteroid sensitivity. However, these findings have yet to be translated into effective therapeutics that improve symptoms and reduce exacerbations in airway disease. This discrepancy can be attributed to several factors, including the lack of optimization in dosing, timing, and route administration. Additionally, antioxidant strategies have not been carefully developed nor optimized to work in combination with corticosteroids. While reducing the oxidative burden may have therapeutic promise, additional investigations are needed to understand the mechanisms related to oxidative stress in the airway and how enhanced antioxidant responses can be leveraged to improve corticosteroid efficacy. We propose that targeting antioxidant responses in the lung remains an appealing strategy to augment corticosteroid sensitivity in airway disease.

## Data Availability

Not applicable.

## Appendix A

**Table A1:** Candidate Compounds to Improve Corticosteroid Sensitivity.

Compound	Disease Model	Experimental Model	Primary Endpoint (s)	Dose	Reference
Antagomir-21 and LY294002	Asthma	Mouse	↑ HDAC2 expression ↓ AHR	2 mg/kg LY294002 50 ug Ant-21 2 mg/kg Dexamethasone	[185]
MCC950 (NLRP3 Inhibitor)	Asthma	Mouse	↓ Neutrophils ↓ AHR ↓ IL-1β levels	10 mg/kg MCC950 2 mg/kg Dexamethasone	[222]
γ-tocotrienol	Asthma	Mouse	↑ Nrf2 expression ↓ AHR ↓ IgE	250 mg/kg γ-tocotrienol 10 mg/kg Prednisolone	[275]
Resveratrol (polyphenol)	Asthma	Mouse	↓ TNFα levels ↓ Mast cell degranulation	50 mg/kg Resveratrol 1 mg/kg Dexamethasone	[280]
Curcumin	COPD	Human monocytes (U937)	↑ HDAC2 expression	1 μM Curcumin 100 nM Dexamethasone or 1 nM budesonide	[283]
N-acetylcysteine (NAC)	Asthma	Mouse	↓ Eosinophils and neutrophils ↓ IL-5 and IL-13 levels	320 mg/kg NAC 1 mg/kg dexamethasone	[284]
Carbocysteine	COPD	Alveolar epithelial cells	↑ HDAC2 expression and activity ↑ Glutathione levels	300 mg/kg Carbocysteine 5 mg/kg Dexamethasone	[287]
Sulforaphane (Nrf2 Agonist)	Asthma	Mouse	↑ Nrf2 activity ↓ Neutrophils ↓ IL-17A and IL-23	25 mg/kg Sulforaphane 1 mg/kg Dexamethasone	[292]
	Asthma/COPD	Mouse	↑HDAC2 activity	12.5 mg/kg Sulforaphane 2.5 mg/kg Dexamethasone	[293]
GYY4137 (H_2_S donor)	COPD	Human monocytes (U937)	↓ TNFα and IL-8 secretion	100–500 μM of GYY4137 10 nM dexamethasone	[304]
Andrographolide	COPD	Human PBMCs and monocytes (U937)	↑ Nrf2 and HDAC2 expression ↓ CXCL8 secretion	1–30 μM Andrographolide 0.1 nM–10 µM Dexamethasone	[305]
Antagomir-9	Asthma	Mouse pulmonary macrophages and human sputum cells	↓ PP2A activity ↓ AHR ↑ GR nuclear translocation	50 mg Antagomir-9 1 mg/kg Dexamethasone	[306]
GW856553 (p38 inhibitor)	COPD	Human PBMCs	↓ CXCL8 and IL-6 secretion ↓ p38 MAPK activity	1 nM GW856553 1 µM dexamethasone	[307]
